# Connexin-45 is expressed in mouse lymphatic endothelium and required for lymphatic valve function

**DOI:** 10.1172/jci.insight.169931

**Published:** 2024-07-18

**Authors:** Michael J. Davis, Jorge A. Castorena-Gonzalez, Min Li, Scott D. Zawieja, Alex M. Simon, Xin Geng, R. Sathish Srinivasan

**Affiliations:** 1 Department of Medical Pharmacology & Physiology, University of Missouri, Columbia, Missouri, USA.; 2 Department of Pharmacology, Tulane University School of Medicine, New Orleans, Louisiana, USA.; 3 Department of Physiology, University of Arizona School of Medicine, Tucson, Arizona, USA.; 4 Cardiovascular Biology Research Program, Oklahoma Medical Research Foundation, Oklahoma City, Oklahoma, USA.

**Keywords:** Cell biology, Vascular biology, Embryonic development, Lymph

## Abstract

The expression and functional relevance of the gap junction molecule connexin-45 (Cx45; *GJC1*) in lymphatic endothelium were not previously known. We found that Cx45 was expressed widely in the endothelium of murine lymphatics, in both valve and nonvalve regions. Cell-specific deletion of Cx45, driven by a constitutive Cre line (*Lyve1-Cre*) or an inducible Cre line (*Prox1-CreER^T2^*), compromised the function of lymphatic valves, as assessed by physiological tests (back leak and closure) of isolated, single-valve vessel segments. The defects were comparable to those previously reported for loss of Cx43, and as with Cx43, deletion of Cx45 resulted in shortening or increased asymmetry of lymphatic valve leaflets, providing an explanation for the compromised valve function. In contrast with Cx43, lymphatic endothelial cell–specific (LEC-specific) deletion of Cx45 did not alter the number of valves in mesenteric or dermal lymphatic networks or the expression patterns of the canonical valve-associated proteins PROX1, ITGA9, or CLAUDIN5. Constitutive deletion of Cx45 from LECs resulted in increased backflow of injected tracer in popliteal networks in vivo and compromised the integrity of the LEC permeability barrier in a subset of collecting vessels. These findings provide evidence for an unexpected role of Cx45 in the development and maintenance of lymphatic valves.

## Abstract 2

Connexin45 (GJC1) is expressed in mouse lymphatic endothelium and required for normal lymphatic valve development and function

## Introduction

A major function of the peripheral lymphatic system is to return excess fluid and protein from the interstitium back to the blood circulation. After absorption into lymphatic capillaries, lymph is transported through lymphatic vessel networks by 2 major mechanisms: 1) the active, spontaneous pumping of collecting lymphatic vessels and 2) the passive compression of lymphatic collectors and capillaries by adjacent tissues (e.g., skeletal muscle). Both mechanisms rely on the proper functioning of 1-way lymphatic valves, interposed at short distances, to prevent the backflow of lymph.

Lymphatic valves (LVs) are thin, highly flexible, bileaflet structures formed by 2 layers of lymphatic endothelial cell (LECs) surrounding a matrix core whose composition includes collagen (1), laminin-5 (2), integrin α9 (2), elastin (3), and fibronectin (2). LVs are typically composed of 2 semicircular leaflets that insert into the vessel wall along their outer edges. The midpoints of each leaflet oppose each other across the vessel wall while the downstream free edges form an elliptical opening in an enlarged sinus area. Valves are normally biased to be open in the absence of a trans-valvular luminal pressure difference (∆P) and gate passively according to the prevailing ∆P. When the leaflets close, there is a variable amount of interleaflet overlap, with an increased area of overlap presumably leading to a tighter seal. The magnitude of the ∆P required for valve closure depends strongly on vessel diameter such that low values of ∆P < 0.3 cmH_2_O (inflow < outflow) are sufficient to close valves when vessel diameter is small, whereas larger values of ∆P are required when vessel diameter is near maximal (4). This relationship between diameter and the ∆P required for valve closure is confirmed by numerical modeling, which predicts that expansion of the vessel lumen at the base and lateral margins of the valve increases tension on the leaflets, requiring more force to close (5).

The current hypothesis for LV formation is that oscillatory shear stress initiates the activation of a gene transcription program involving *Prox1, Gata2, Foxc2*, and other factors (6–9). *Prox1* is critical for LEC specification and *Foxc2* for the initiation of valve formation (10). Selective deletion of *Foxc2* from LECs in late embryonic or early postnatal stages results in reduced LV density and a significant percentage of valves with severe back leak (7). *Foxc2*-haplodeficient mice have normal viability. However, the number of LVs is reduced by approximately 50%, and many of the remaining valves have slight back leak (11, 12). Numerous other regulators of LEC growth or matrix production (13) have been implicated in LV formation or maintenance, but it remains to be defined how they interact to produce functionally normal LVs. In addition to the regulators mentioned above, several LEC-LEC junctional molecules are critical for LV development or maintenance, including VE-cadherin (14, 15) and at least 3 connexin (Cx) isoforms (16–18). Missense mutations in Cx43 (*GJA1*) and Cx47 (*GJC2*) are each linked to the development of lymphedema in humans (19–21), and women with mutations in *GJC2* have an increased likelihood of developing lymphedema after breast cancer surgery (22).

In a previous study, images of Cx45 staining in the lymphatic muscle cell (LMC) layer of mouse and human collecting lymphatics revealed additional faint Cx45 signal in axially oriented cells underneath the LMC layer (23), suggesting that Cx45 might also be expressed in lymphatic endothelium. The goal of the present study was to assess whether Cx45 is indeed expressed in LECs and, if so, to test the consequences of Cx45 deficiency on LV development and function. Here, we identified Cx45 expression in LECs throughout the popliteal, mesenteric, and dermal lymphatic networks, in both valve and nonvalve regions. Expression was verified in popliteal lymphatics by PCR analysis and by LEC-specific expression of GFP driven by the endogenous Cx45 promoter (24, 25). In vivo tests using *Lyve1-Cre Cx45*^Δ/fl^ mice revealed backflow of injected dye into side branches of popliteal lymphatic collectors as well as perilymphatic dye leakage. Ex vivo tests of valve function in control and Cx45-deficient popliteal lymphatic vessels indicated that Cx45 deletion in developing LVs, using a constitutive *Lyve1-Cre*, resulted in back leak defects and an increased percentage of valves with abnormal back leak. Deletion of Cx45 after LV development was complete, using an inducible *Prox1-CreER^T2^*, resulted in a significant increase in the fraction of LVs with back leak. Thus, Cx45 is a previously unidentified LEC Cx isoform that contributes markedly to the development and, to a lesser extent, maintenance of functional LVs.

## Results

**Figure 1:**
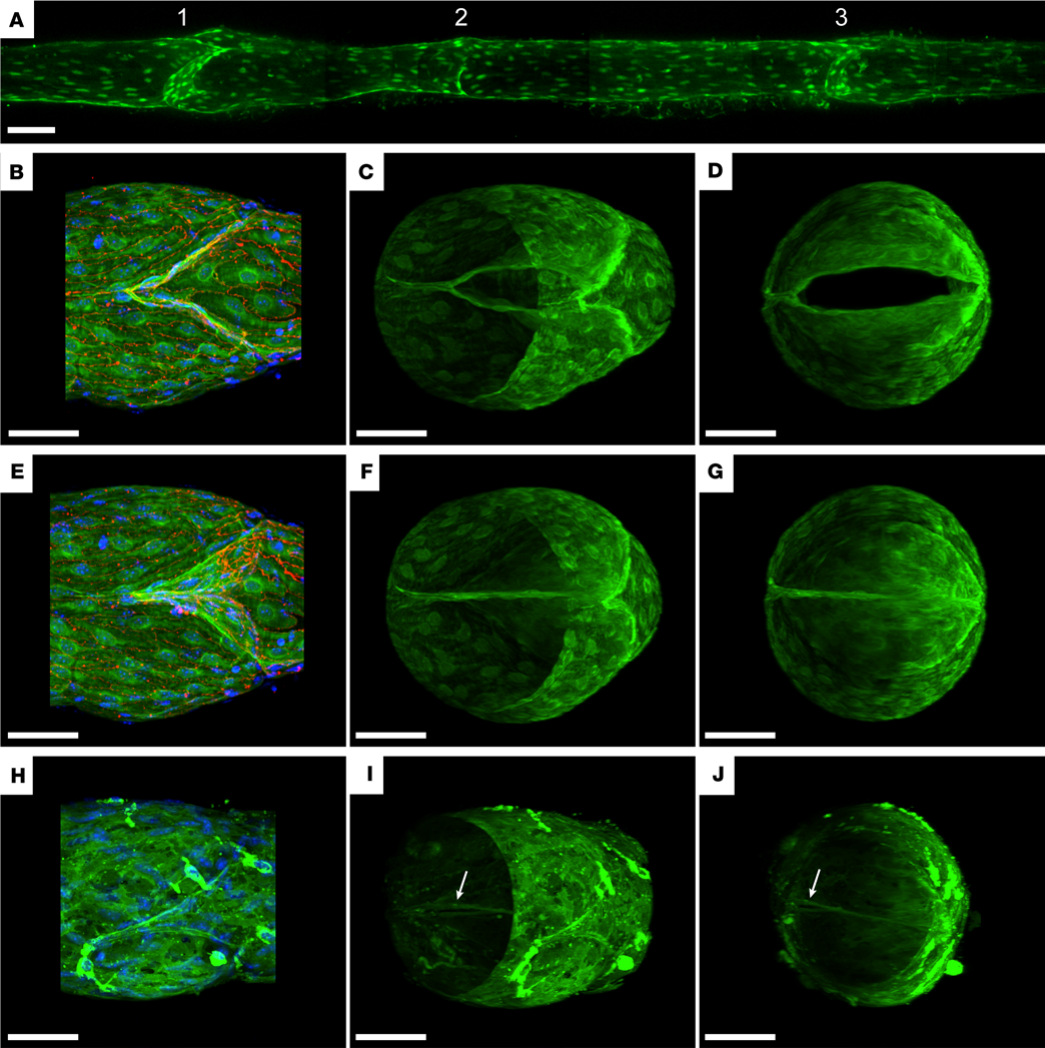
Cx45 is expressed in LECs of popliteal afferent lymphatics. (**A**) Live GFP fluorescence signal (maximum projection) in a 3-valve popliteal lymphatic vessel segment from a *Prox1-CreER^T2^ Cx45^fl/fl^* mouse. The normal direction of flow is left to right. The mouse was approximately 2 months old when induced with tamoxifen. (**B**–**G**) Orthographic isometric projections from 3-dimensional reconstructions of confocal *Z*-stacks from a *Prox1-GFP* vessel with the valve in an open position (**B**–**D**) or closed position (**E**–**G**). The normal direction of flow is right to left. (**H**–**J**) Orthographic isometric projections from 3-dimensional reconstructions of confocal *Z*-stacks from a *Lyve1-Cre Cx45*^Δ/fl^ vessel with a valve displaying deficient seal in the presence of an adverse pressure gradient. Note the small gap between the 2 closed leaflets near the insertion point in **I** and **J** (indicated with arrows). Vessel segments were stained with an anti-GFP antibody (green) and DAPI (blue). Panels **B** and **E** also display staining of VE-cadherin (red). Scale bars are 50 μm.

Excision of the floxed Cx45 sequence using *Prox1-CreER^T2^* resulted in GFP expression (24) in popliteal lymphatics isolated from 1- to 2-month-old mice. GFP-positive LECs were prominent along the entire vessel length, in both valve and nonvalve regions, as evident from live fluorescence images of cannulated, pressurized vessels from *Prox1-CreER^T2^ Cx45^fl/fl^* mice. Of the 3 valves in the vessel segment in Figure 1A, oriented with the normal flow direction from left to right, valves 1 and 3 appeared to have normal-shaped sinuses and normal-length leaflets, but valve 2 had a relatively indistinct sinus and somewhat short leaflets. Similar GFP fluorescence patterns in LECs were evident in lymphatic collectors from other regions of *Prox1-CreER^T2^ Cx45^fl/fl^* mice, including superior cervical lymphatics, inguinal-axillary lymphatics, and mesenteric lymphatics (not shown).

Popliteal valves in vessels from *Prox1-GFP* and *Lyve1-Cre Cx45*^Δ/fl^ mice were imaged using confocal microscopy to obtain a higher resolution of the leaflet structure, and examples are shown in Figure 1, B–J, each oriented with the normal flow direction from right to left. For confocal imaging, the vessels were pressurized and fixed overnight with 1% paraformaldehyde (PFA) **(**prior to immunostaining with an anti-GFP antibody. After deconvolution of each image, a maximal projection of the reconstructed *z* axis image stacks was generated. Views of a valve from a *Prox1-GFP* mouse with the leaflets open are shown in Figure 1, B–D, and were obtained by setting inflow pressure (Pin) ≥ outflow pressure (Pout); views of the same valve with the leaflets closed were obtained by setting Pout > Pin (Figure 1, E and F). Views of another closed valve from a *Lyve1-Cre Cx45*^Δ/fl^ mouse are shown in Figure 1, H–J, in which GFP is expressed in both LECs and macrophages and in which a small gap (at arrows in Figure 1, I and J) is evident at the left margin where the leaflets insert into the wall. Although the gap could be an indication of a partially defective valve, in this case it was more likely an artifact of fixation, because valve function tests on this same valve before fixation required an adverse pressure gradient (ΔP; Pout–Pin) < 0.5 cmH_2_O for closure compared with a ΔP > 5 cmH_2_O after fixation. Rotatable movies of these confocal stacks are provided in Supplemental Videos 1 and 2; supplemental material available online with this article; https://doi.org/10.1172/jci.insight.169931DS1 These results show that Cx45 is expressed in LECs and suggest that mice deficient in *Cx45* exhibit both morphologically normal and abnormal LVs.

**Figure 2:**
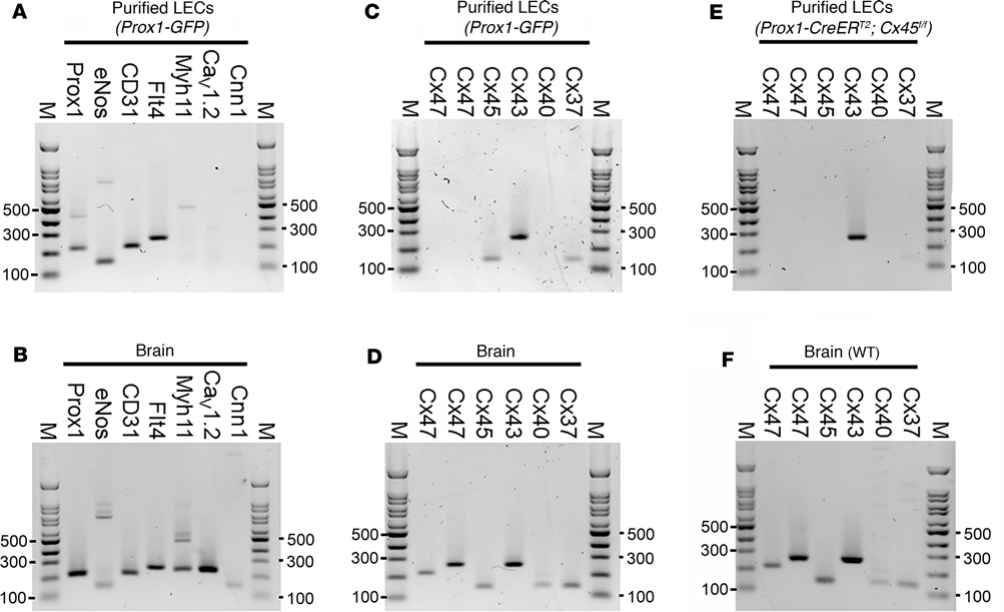
PCR evidence for expression of Cx43, Cx45, and Cx37 in LECs of popliteal afferent lymphatics and the effective deletion of Cx45 using *Lyve1-Cre*. (**A** and **B**) PCR of FACS-purified LECs sorted from inguinal-axillary lymphatics from *Prox1-GFP* mice (**A**) showing expression of LEC genes but not LMC genes, with brain homogenate (**B**) serving as a positive control for all primers. (**C**) Cx45, Cx43, and Cx37 expression in purified LECs (**C**) and in brain homogenate (**D**). Two different Cx47 primers were used. (**E**) *Prox1-CreER^T2^ Cx45^fl/fl^* vessels expressed Cx43 and Cx37 (faint signal) but lacked Cx45. (**F**) Brain homogenate served as a positive control for primers in **E**. All gels are representative of at least 3 similar experiments. Myh11, myosin heavy chain 11; Cav1.2, L-type voltage-gated calcium channel; Cnn1, Calponin1.

PCR analysis was used to check for Cx45 mRNA in native LECs. For this analysis inguinal-axillary lymphatic vessels were used because they contained more cells than popliteal vessels. Since Cx45 is highly expressed in LMCs (23), the LEC population was purified before performing PCR. Inguinal-axillary lymphatic vessels were dissected from multiple *Prox1-GFP* mice and carefully cleaned to remove any attached small blood vessels or capillaries. After enzymatic digestion to obtain single cells (~5,000 cells/vessel), the cell suspension was purified by FACS, sorting on the GFP signal, and then probed for known LEC and LMC markers. As expected, the preparation showed message for the LEC markers Prox1, eNOS, CD31, and Flt4 but was negative for the canonical LMC markers Myh11, Ca_V_1.2, and Cnn1, as shown in Figure 2A (see Table 1 for a list of primer sequences, gene accession numbers, and amplicons). The purified LEC lysate from similarly obtained lymphatics was probed for the major vascular Cx isoforms previously detected in whole lymphatic vessels (23). mRNA was detected for Cx45, Cx43, and Cx37, but not for Cx47 or Cx40 (Figure 2C). The absence of Cx47 expression, despite the use of 2 different primers, is puzzling but in agreement with analysis of whole inguinal-axillary lymphatic vessels in a previous study (23). It is possible that Cx47 detected in earlier studies (26, 27) may be explained if its expression is restricted to a certain developmental stage or to lymphatic vessels from certain regions or to valve leaflets; in the latter case, our detection method would not be sufficiently sensitive. Samples of mouse brain tissue served as positive controls for the primers used in these assays (Figure 2, B and D). Deletion of Cx45 was verified by analysis of FACS-purified LECs from inguinal-axillary lymphatic vessels of *Prox1-CreER^T2^ Cx45^fl/fl^* mice, where the expression of Cx43 and Cx37 (although faint) continued to be detected in the absence of message for Cx45 (Figure 2E, with positive controls for primers shown in Figure 2F). For the latter analysis *Prox1-GFP* rather than *Cx45^fl/fl^* vessels were used as the reference sample, because a GFP signal was needed to sort and purify the control LEC population. See Supplemental Figure 1 for the gating windows used in the FACS analysis.

Subsequently, quantitative real-time PCR (qPCR) was performed on FACS-sorted LECs from *Prox1-GFP* and *Prox1-CreER^T2^ Cx45^fl/fl^* mice to quantitatively test if Cx45 deletion resulted in upregulation of other lymphatic Cx isoforms (Supplemental Figure 2). As expected, Cx45 message was reduced to less than 10% of control in LECs from *Prox1-CreER^T2^ Cx45^fl/fl^* vessels while Cx43 and Cx37 mRNA levels were not significantly different between LECs from *Prox1-GFP* and *Prox1-CreER^T2^ Cx45^fl/fl^* vessels. The signals for Cx47 were too low for meaningful analysis. Thus, deletion of Cx45 did not result in significant compensatory upregulation in the mRNAs of other LEC connexins.

**Figure 3:**
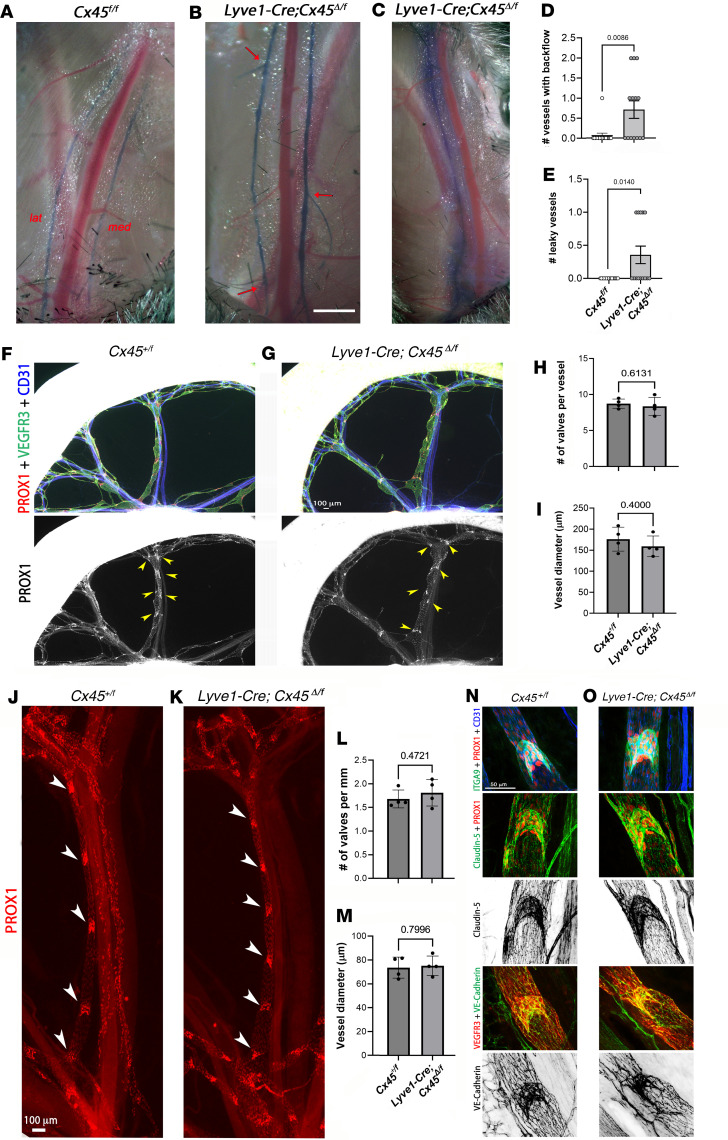
Constitutive Cx45 deletion leads to EBD backflow and leakage but does not interfere with the development of mesenteric LVs. (**A** and **B**) Popliteal afferent lymphatics in the intact hind limbs of *Cx45^fl/fl^* and *Lyve1-Cre Cx45^fl/fl^* mice after footpad injection of EBD. Bottoms of images are toward feet. (**A**) Dye is contained in both *Cx45^fl/fl^* vessels, but (**B**) backflow into side branches occurs at 3 sites (red arrows) in 2 *Lyve1-Cre Cx45*^Δ/fl^ vessels. Scale bar: 1 mm. LAT, lateral; MED, medial. (**C**) One *Lyve1-Cre Cx45*^Δ/fl^ popliteal afferent was leaky without any backflow. (**D**) The number of side branches (per popliteal afferent) with backflow after EBD injection. (**E**) The number of popliteal lymphatics that leaked EBD. Means ± SEM. Significant differences between *Cx45^fl/fl^* and *Lyve1-Cre Cx45*^Δ/fl^ vessels assessed using Mann-Whitney *U* test. *N* = 4, *n* = 14 for *Lyve1-Cre Cx45*^Δ/fl^ *N* = 4, *n* = 16 for *Cx45^fl/fl^*. (**F** and **G**) Mesenteric LVs from E18.5 *Cx45^+/fl^* and *Lyve1-Cre Cx45*^Δ/fl^ embryos were morphologically normal. Mesenteric arcades from *Cx45^+/fl^* and *Lyve1-Cre Cx45*^Δ/fl^ embryos were stained using indicated antibodies. The number of LVs per vessel (yellow arrowheads in **F** and **G**) was counted and quantified. (**H** and **I**) Neither the number of valves per vessel nor vessel diameter was affected by deletion of *Cx45*. *N* = 4 pups/genotype. Mean ± SD with each dot representing an embryo. Unpaired, 2-tailed *t* tests were used for statistical analysis. (**J** and **K**) The mesenteries of *Cx45^+/fl^* and *Lyve1-Cre Cx45*^Δ/fl^ P10 pups were stained for Prox1 (white arrowheads indicate valves). No significant differences were observed in either the number of valves per vessel length (**L**) or vessel diameter (**M**). Mean ± SD. Unpaired, 2-tailed *t* tests were used for statistical analysis. *N* = 4 pups/genotype. (**N** and **O**) The expression patterns of INTEGRIN-α9, CLAUDIN-5, and VE-CADHERIN were indistinguishable between *Cx45^+/fl^* and *Lyve1-Cre Cx45*^Δ/fl^ P10 mesenteric valves. *N* = 4 pups/genotype.

Next, in vivo evidence for backflow was examined in lymphatic networks of control and Cx45-deficient mice. Evan’s Blue Dye (EBD) was injected into the foot pads of anesthetized *Cx45^fl/fl^* or *Lyve1-Cre Cx45*^Δ/fl^ mice and allowed to fill the popliteal lymphatic network. The skin over the dorsal-medial surface of the thigh in each leg was opened and retracted to expose the 2 dye-filled popliteal afferent lymphatic vessels. As shown in Figure 3A, both popliteal vessels were visible on either side of the saphenous vein of a *Cx45^fl/fl^* mouse with no indication of EBD backflow into any side branches. Under similar conditions in a *Lyve1-Cre Cx45*^Δ/fl^ mouse, backflow was evident in 2 side branches of the lateral popliteal afferent and 1 side branch of the medial popliteal afferent (Figure 3B). We also noted that a subset (5 of 14) of popliteal vessels in *Lyve1-Cre Cx45*^Δ/fl^ mice appeared to be leaky, as evident in the lateral vessel (left side of the vein) in Figure 3C. The number of popliteal afferents with backflow into side branches and the number of leaky popliteal vessels were quantified and plotted in Figure 3, D and E. There were significant increases in both the number of vessels with backflow and the number of leaky vessels in *Lyve1-Cre Cx45*^Δ/fl^ mice compared with *Cx45^fl/fl^* mice.

The occurrence of backflow in vivo and vessel images such as those shown in Figure 1, A and J, suggested possible LV defects in Cx45-deficient lymphatic vessels. Therefore, LV development was characterized in *Lyve1-Cre Cx45*^Δ/fl^ mice at embryonic and neonatal stages in the mesentery. Examination of the mesenteries of E18.5 embryos revealed no obvious reductions in the number of valves in *Lyve1-Cre Cx45*^Δ/fl^ vessels, compared with vessels from *Cx45^+/fl^* mice, based on the number of Prox1-high nuclei clusters present in mesenteric lymphatic networks in situ (Figure 3, F and G). Quantification revealed no significant differences from control vessels in the number of mesenteric valves per vessel at E18.5 (Figure 3H). The diameters of mesenteric lymphatic vessels were similar between the 2 strains (Figure 3I), indicating that there was no collecting vessel hyperplasia at E18.5. Mesenteries from P10 pups were stained for Prox1 (Figure 3, J and K); no significant differences were observed in the Prox1 staining pattern or the number of valves per vessel length (Figure 3L) or lymphatic vessel diameter (Figure 3M). *Cx45* deletion using *Lyve1-Cre* did not interfere with the expression of the canonical LEC valve proteins Prox1, Vegfr3, and Itga9, as the immunostaining patterns of these proteins were similar in mesenteric lymphatic vessels from *Cx45^+/fl^* and *Lyve1-Cre Cx45*^Δ/fl^ P10 pups (Figure 3, N and O). Likewise, *Cx45* deletion did not appear to affect the expression patterns of the tight junction molecule Claudin-5 or the adherens junction molecule VE-cadherin (Figure 3, N and O). We also checked for defects in the lymphovenous valves (LVVs). LVVs are the entry points for lymph into the venous system, the first valves to develop outside the heart (28), and valves in which defects are often associated with lymphatic disorders (29). There were no obvious defects in the morphology of the LVVs at E16.5 in *Lyve1-Cre Cx45*^Δ/fl^ mice or in the immunostaining pattern for the valve-regulatory molecule Prox1 in LVVs (Supplemental Figure 3, A and B, arrows). The venous valves that develop in the vicinity of LVVs also appeared to be normal (Supplemental Figure 3, A and B, arrowheads).

*Lyve1-Cre Cx45*^Δ/fl^ mice did not exhibit any obvious systemic pathologies, any decrease in viability, or the chylous ascites or chylothorax previously reported in mice with deficiencies in Cx43 or Cx37 (26). Collectively, these results suggest that constitutive deletion of *Cx45* does not alter the number of valves in the mesenteric lymphatic network or the gross morphology or expression patterns of canonical LEC valve proteins in LVs and LVVs, though this analysis does not rule out subtle differences in valve leaflet structure or function. The leak of EBD in some vessels, as shown in Figure 3, C and E, suggests that a subtle permeability defect may not be reflected in the immunostaining patterns of Claudin-5 and VE-cadherin, may be indicative of the requirement of Cx45 in maintaining vessel integrity during adulthood but not at neonatal stages, or may reflect differences between the roles of Cx45 in LECs of mesenteric versus popliteal lymphatic vessels.

Tests of valve function (30) were then performed on popliteal lymphatics from *Lyve1-Cre Cx45*^Δ/fl^ mice, in which Cx45 was deficient during development, and from *Prox1-CreER^T2^ Cx45^fl/fl^* mice, in which LVs were allowed to develop normally followed by postnatal deletion of *Cx45* P30–P60. Low-pressure back leak tests were used to measure the degree of valve competency when pressures were within the presumed normal range for mice (0.5–10 cmH_2_O). Closure tests were used to detect possible changes in valve stiffness, in which higher-than-normal adverse pressure gradients would be needed to close the valve. All valve function tests were conducted on single-valve popliteal lymphatic segments ex vivo, with spontaneous contractions eliminated.

**Figure 4:**
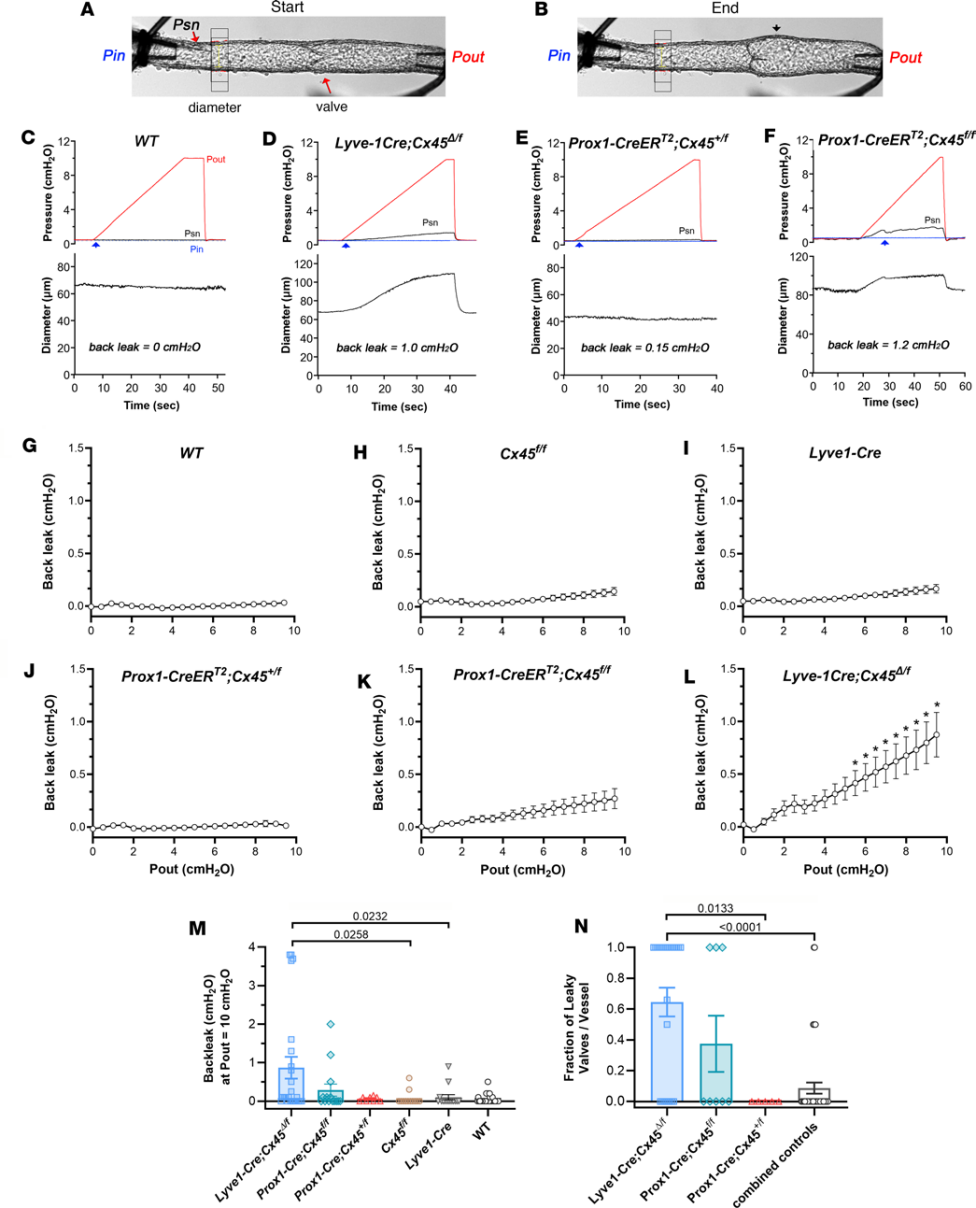
Back leak tests. Images of a popliteal lymphatic containing a single valve (**A**) before and (**B**) after elevation of Pout to 10 cmH_2_O. Black arrow points to expanded valve sinus. (**C**–**F**) Representative examples of back leak tests for valves from (**C**) *WT*, (**D**) *Lyve1-Cre Cx45*^Δ/fl^, (**E**) *Prox1-CreER^T2^ Cx45^+/fl^*, and (**F**) *Prox1-CreER^T2^ Cx45^fl/fl^* mice. Arrowheads indicate valve closure. Values of back leak (Psn–Pin) at Pout = 10 cmH_2_O are stated above the traces. (**G**–**L**) Summary of back leak measurements as a function of Pout for each of the 6 genotypes. The raw Psn data were binned in 0.5 cmH_2_O Pout intervals before determining the mean and variance of Psn for each interval. Asterisks in **L** indicate significant differences (*P* < 0.05) for *Lyve1-Cre Cx45*^Δ/fl^ valves compared to their initial (control) values at Pout levels > 5 cmH_2_O, as determined by a 2-way, repeated measures ANOVA with Tukey’s post hoc tests. None of the other groups had significant increases in Psn. (**M**) Comparisons of back leak at Pout = 10 cmH_2_O between genotypes. *Lyve1-Cre Cx45*^Δ/fl^ and *Lyve1-Cre Cx45^fl/fl^* data were combined. Comparisons between *Lyve1-Cre Cx45*^Δ/fl^, *Lyve1-Cre*, and *Cx45^fl/fl^* valves and between *Prox1-CreER^T2^ Cx45^fl/fl^*, *Prox1-CreER^T2^ Cx45^+/fl^*, and *Cx45^fl/fl^* valves were made using Kruskal-Wallis tests with Dunn’s post hoc tests. Back leak was significantly higher in *Lyve1-Cre Cx45*^Δ/fl^ valves compared with *Lyve1-Cre* or *Cx45^fl/fl^* valves. (**N**) Fraction of leaky valves for each genotype calculated using the threshold criteria defined in text. Comparisons between *Lyve1-Cre Cx45*^Δ/fl^, *Prox1-CreER^T2^ Cx45^fl/fl^*, *Prox1-CreER^T2^ Cx45^+/fl^*, and the combined control groups were made using a Kruskal-Wallis test with Dunn’s post hoc tests. Differences between *Lyve1-Cre Cx45*^Δ/fl^ valves and combined controls and between *Lyve1-Cre Cx45*^Δ/fl^ and *Prox1-CreER^T2^ Cx45^+/fl^* valves were statistically significant. Comparisons not indicated were not significant at *P* < 0.05. In panels **G**–**N**, data are means ± SEM. *WT* *N* = 16, *n* = 32; *Lyve1-Cre Cx45*^Δ/fl^ and *Lyve1-Cre Cx45^fl/fl^* combined *N* = 9, *n* = 24; *Lyve1-Cre* *N* = 6, *n* = 14; *Cx45^fl/fl^* *N* = 4, *n* = 12; *Prox1-CreER^T2^ Cx45^fl/fl^* *N* = 4, *n* = 14; *Prox1-CreER^T2^ Cx45^+/fl^* *N* = 2, *n* = 8. Psn, servo-null pressure.

For back leak tests, Pin was held constant at 0.5 cmH_2_O during measurement of pressure on the upstream side of the valve using a servo-null micropipette. With the servo-null pipette tip in place, Pout was raised ramp-wise from 0.5 to 10 cmH_2_O over the course of about 40 seconds. Diameter was also measured on the upstream side of the valve near the Pin pipette (11). Normal valves closed when Pout exceeded Pin by a fraction of a cmH_2_O (typically <0.3 cmH_2_O at this level of Pin). Images of a 1-valve popliteal lymphatic segment are shown in Figure 4 at the start (Figure 4A) and end (Figure 4B) of the test and illustrate how only the downstream half of the vessel distends at Pout = 10 cmH_2_O (Figure 4B, arrowhead) while any pressure increase in the upstream half is prevented by a competent, closed valve. Representative recordings of low-pressure back leak tests are shown in Figure 4, C–F, for 4 genotypes of mice: *WT* (C57BL/6), *Lyve1-Cre Cx45*^Δ/fl^*,* *Prox1-CreER^T2^ Cx45^+/fl^* (haplodeficient in Cx45), and *Prox1-CreER^T2^ Cx45^fl/fl^*. The *WT* valve behaved normally (Figure 4C), with the valve closing as Pout exceeded approximately 0.2 cmH_2_O and with no detectable change in Psn at Pout = 10 cmH_2_O (i.e., Psn–Pin = 0 cmH_2_O). In contrast, the valve in the *Lyve1-Cre Cx45*^Δ/fl^ vessel exhibited some back leak (Psn–Pin = 1.0 cmH_2_O) at the maximal Pout level (Figure 4D) but well short of the theoretical maximum back leak of approximately 4.75 cmH_2_O (halfway between Pout = 10 cmH_2_O and Pin = 0.5 cmH_2_O, with additional variability contributed by the relative resistances of the vessel segments between the pipette tips and the valve). Diameter also increased on the inflow side as Pout rose, consistent with back leak through the closed valve. A similar amount of back leak was recorded in the *Prox1-CreER^T2^ Cx45^fl/fl^* vessel (Figure 4F), but only a minute amount of back leak was evident in the *Prox1-CreER^T2^ Cx45^+/fl^* vessel (Figure 4E).

The data sets for back leak included 3 groups of control vessels — *WT* (C57BL/6J, the background strain for *Prox1-CreER^T2^*), *Lyve1-Cre* (no floxed gene), and *Cx45^fl/fl^* (no Cre) — as well as 3 groups of *Cx45*-deficient vessels: *Lyve1-Cre Cx45*^Δ/fl^, *Prox1-CreER^T2^ Cx45^fl/fl^,* and *Prox1-CreER^T2^ Cx45^+/fl^*. To analyze the back leak data, the individual sets of Psn values during Pout ramps from 0.5 to 10 cmH_2_O were averaged over 0.5 cmH_2_O Pout intervals by binning the raw Psn data and computing the mean and variance for each interval (Figure 4, G–L). For statistical analysis of that data, repeated measures 2-way ANOVAs with Tukey’s post hoc tests compared the back leak data within each group with the control value at the start of the Pout ramp. The analyses revealed significantly elevated levels of back leak for *Lyve1-Cre Cx45*^Δ/fl^ at Pout levels above 5 cmH_2_O.

For statistical comparisons between the genotypes, the single value of back leak recorded at the end of the ramp (Pout = 10 cmH_2_O) was used for each vessel (Figure 4M). Of 17 *Lyve1-Cre Cx45*^Δ/fl^ and 10 *Lyve1-Cre Cx45^fl/fl^* vessels studied (data were combined after a Mann-Whitney *U* test revealed no significant differences between the 2 groups, hereafter referred to as *Lyve1-Cre Cx45*^Δ/fl^ mice), 17 exhibited back leak within the “normal” range, 6 showed intermediate back leak, and 4 were essentially incompetent (i.e., back leak > 3.7 out of an estimated maximal 4.75 cmH_2_O). Of 14 *Prox1-Cre Cx45^fl/fl^* valves, 11 exhibited no back leak and 3 exhibited intermediate back leak. One of 8 valves tested 6–7 weeks after induction was leaky, compared with 2 of 6 valves tested 18 weeks after induction, suggesting a possible trend for these valves to become leakier with time. Not all the control data sets were normally distributed, so nonparametric tests were used. Kruskal-Wallis tests with Dunn’s multiple comparisons post hoc tests compared differences in back leak between *Lyve1-Cre Cx45*^Δ/fl^, *Lyve1-Cre*, and *Cx45^fl/fl^* valves and between *Prox1-Cre Cx45^fl/fl^*, *Prox1-Cre Cx45^+/fl^*, and *Cx45^fl/fl^* valves. The results revealed that back leak was significantly elevated only in *Lyve1-Cre Cx45*^Δ/fl^ valves, suggesting that constitutive deletion of Cx45 from LECs induced back leak defects.

As an alternative analysis of the back leak data, we calculated the fraction of leaky valves for each multivalve segment that was initially isolated and cleaned (Figure 4N). The numbers were more limited and the data were more variable because neither the number of valves in the segment nor the success rate of the valve tests for each vessel could be controlled. Therefore, to strengthen this analysis, the data for the 3 “control groups” (*WT* + *Cx45^fl/fl^* + *Lyve1-Cre*) were combined. As stated in Methods, *Cx45^fl/fl^* and *Lyve1-Cre* mice were backcrossed to *WT* mice so that the 3 strains were congenic. We first verified, using a Kruskal-Wallis test, that there were no significant differences in back leak between the 3 strains. We then defined a “leaky” valve as having back leak higher than the mean + 1 SD of the pooled 3 control groups; this value equaled 0.24 cmH_2_O at Pout = 10 cmH_2_O. The fraction of valves in the unshortened vessel segment with back leak higher than this level were then classified as leaky. This analysis concurred with the data in Figure 4M in showing that the combined *Lyve1-Cre Cx45*^Δ/fl^ + *Lyve1-Cre Cx45^fl/fl^* vessels had a significantly higher percentage of leaky valves than the combined control vessels and a significantly higher percentage of leaky valves than *Prox1-CreER^T2^ Cx45^+/fl^* valves. Collectively, these analyses show that constitutive deletion of Cx45 from LECs consistently induced back leak defects compared with control valves or valves with inducible deletion of Cx45 from LECs.

**Figure 5:**
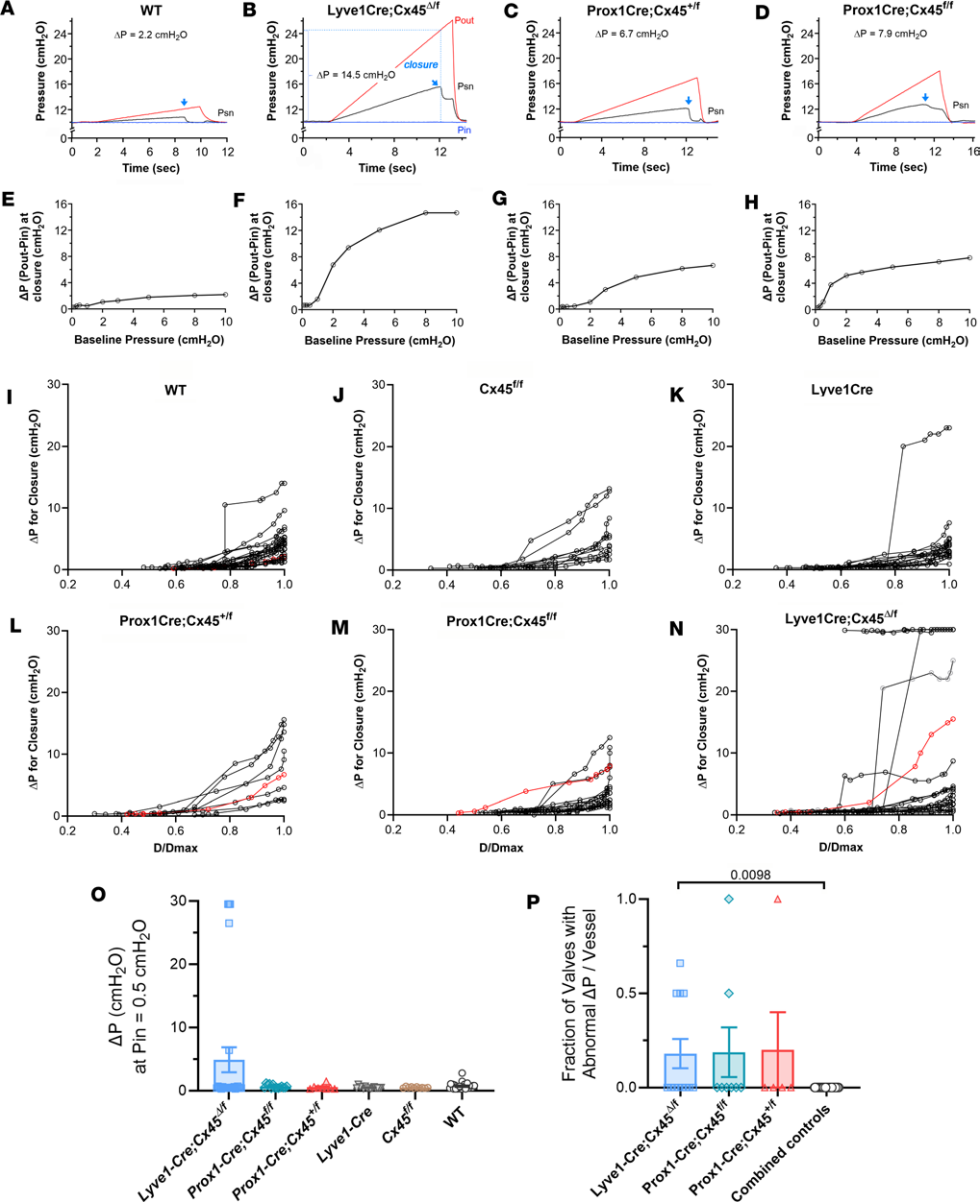
Closure tests. Representative recordings of closure tests for valves from (**A**) *WT*, (**B**) *Lyve1-Cre Cx45*^Δ/f^, (**C**) *Prox1-CreER^T2^ Cx45^+/fl^*, and (**D**) *Prox1-CreER^T2^ Cx45^fl/fl^* mice at baseline pressure Pin = 10 cmH_2_O. Arrows indicate when the valve closed during the ramp. (**B**) ΔP from Pout–Pin at valve closure. (**E**–**H**) Complete back leak curves versus baseline pressures for the same valves. Filled red circles representing ΔP values in top panels. (**I**–**N**) Complete closure tests for the 6 genotypes of mice. Red symbols/curves are data shown in panels **E**–**H**. Normal valves had a ΔP for closure <10 cmH_2_O when vessel diameter was maximal. Three *Lyve1-Cre Cx45*^Δ/fl^ valves were completely incompetent at all pressures, and a fourth valve became incompetent when diameter exceeded 70% of the maximal diameter. (**O**) Comparisons of ΔP for closure values at Pin = 0.5 cmH_2_O for the 6 genotypes. A Kruskal-Wallis test was used to determine significant differences for each genotype compared with the control groups. No significant differences were found. (**P**) Alternative analysis comparing the fraction of vessels with abnormal ΔP for closure at Pin = 0.5 cmH_2_O between the different genotypes. Because of the higher variability in this parameter between vessels, the control groups (*WT* + *Lyve1-Cre* + *Cx45^fl/fl^*) were combined, and the threshold for classifying a valve as abnormal was determined from the mean + 1 SD of the combined controls. A Kruskal-Wallis test with Dunn’s post hoc tests showed a significant difference between *Lyve1-Cre Cx45*^Δ/fl^ valves and the combined controls. Means ± SEM. In panel **O**, the values of *N* and *n* are the same as in Figure 4. In panel **P**, *WT* *N* = 16, *n* = 24; *Lyve1-Cre Cx45*^Δ/fl^ and *Lyve1-Cre Cx45^fl/fl^* combined *N* = 9, *n* = 12; *Lyve1-Cre* *N* = 6, *n* = 10; *Cx45^fl/fl^* *N* = 4, *n* = 6; *Prox1-CreER^T2^ Cx45^fl/fl^* *N* = 4, *n* = 8; *Prox1-CreER^T2^ Cx45^+/fl^* *N* = 2, *n* = 5.

Next, we analyzed closure tests performed on the same valves. Recall that closure tests reflect the stiffness of the valve leaflets rather than the degree to which the leaflets seal. Representative examples of closure tests (all at a single value of Pin, 10 cmH_2_O) are shown in Figure 5. The protocol and associated measurements are illustrated by the recording for the *Lyve1-Cre Cx45*^Δ/fl^ vessel in Figure 5B. As Pout rose, the valve remained open (with increasing backflow) until it closed at Pout ~24 cmH_2_O, marked by a sharp drop in Psn, which had risen from 10 to 15.8 cmH_2_O. At this moment the value of Pout–Pin (the ΔP for closure) was 14 cmH_2_O. The closure test for the *WT* valve was indicative of a normal valve in that the valve closed when ΔP was ~1 cmH_2_O, and Psn instantly returned from 11 to its baseline value of 10 cmH_2_O (Figure 5A). The observation that Psn did not fall back to 10 cmH_2_O in the *Lyve1-Cre Cx45*^Δ/fl^ vessel when the valve closed (Figure 5B) is further evidence that there was significant pressure back leak through that closed valve (back leak is even more obvious in this test than the test at the lower value of Pin = 0.5 cmH_2_O shown in Figure 4D). Closure tests for *Prox1-CreER^T2^ Cx45^+/fl^* and *Prox1-CreER^T2^ Cx45^fl/fl^* valves showed values of ΔP for closure that were intermediate between those of the *WT* valve and the *Lyve1-Cre Cx45*^Δ/fl^ valve. These tests were repeated over the presumed physiological pressure range for mouse lymphatics (from 0.1 to 10 cmH_2_O), allowing the generation of the curves shown in panels of Figure 5, E–H, which characterized the complete relationship between the ΔP for closure and the baseline pressure, with each valve described by a concave curve. The curve for the *WT* valve was similar to *WT* curves described in previous studies (4, 31), with a maximal value of ΔP ~2 cmH_2_O. For our standard analysis, ΔP for closure was plotted as a function of normalized diameter, rather than the raw Pin value, resulting in sets of upward convex curves for each valve studied, as shown in Figure 5, I–N. The red symbols/lines indicate the data for the 4 valves shown in Figure 5, E–H. The closure curves for 3 of the *Lyve1-Cre Cx45*^Δ/fl^ (combined with *Lyve1-Cre Cx45^fl/fl^*) valves were notably distinct (Figure 5N, red arrow) as represented by flat curves at ΔP = 30 cmH_2_O, indicating that these valves were completely incompetent and incapable of closing at the maximum pressure gradients imposed. Three other *Lyve1-Cre Cx45*^Δ/fl^ valves became incompetent or nearly incompetent after diameter exceeded approximately 70% of maximum diameter (Dmax) (one was the vessel shown in Figure 5B). No valves in any of the other groups exhibited this behavior, though 1 *Lyve1-Cre* valve had an abnormally high ΔP for closure as normalized diameter exceeded 0.8.

The sets of ΔP values for closure versus D/Dmax curves were difficult to compare statistically because of continuous *x* and *y* axes; therefore, the single value of ΔP at a given physiological Pin level (0.5 cmH_2_O) was used for comparisons between genotypes. Not all data sets were normally distributed, so nonparametric tests were used. Kruskal-Wallis tests with Dunn’s multiple comparisons tests were used to compare differences in the ΔP for closure between *Lyve1-Cre Cx45*^Δ/fl^, *Lyve1-Cre*, and *Cx45^fl/fl^* valves and between *Prox1-CreER^T2^ Cx45^fl/fl^*, *Prox1-CreER^T2^ Cx45^+/fl^*, and *Cx45^fl/fl^* valves (Figure 5O). Despite the incompetent *Lyve1-Cre Cx45*^Δ/fl^ valves noted above, the differences between the genotypes did not reach statistical significance. As an alternative analysis, we calculated the fraction of popliteal vessels with an abnormal ΔP required for closure at Pin = 0.5 cmH_2_O and compared the Cx45-deficient valves with the combined control (*WT* *+* *Cx45^fl/fl^* *+* *Lyve1-Cre*) valves. Similar to the back leak analysis, the threshold for determining that a valve had an abnormal ΔP for closure was calculated from the mean + 1 SD of the combined 3 control groups (threshold ΔP = 1.02 cmH_2_O). Significant differences were noted in the percentage of LVs with abnormally high ΔP values for *Lyve1-Cre Cx45*^Δ/fl^ compared with the combined controls. Collectively, the results in Figure 5, O and P, suggest that a subset of *Lyve1-Cre Cx45^fl/fl^* valves were incompetent and that deletion of Cx45 interfered with the ability of LVs to close under an adverse pressure gradient.

**Figure 6:**
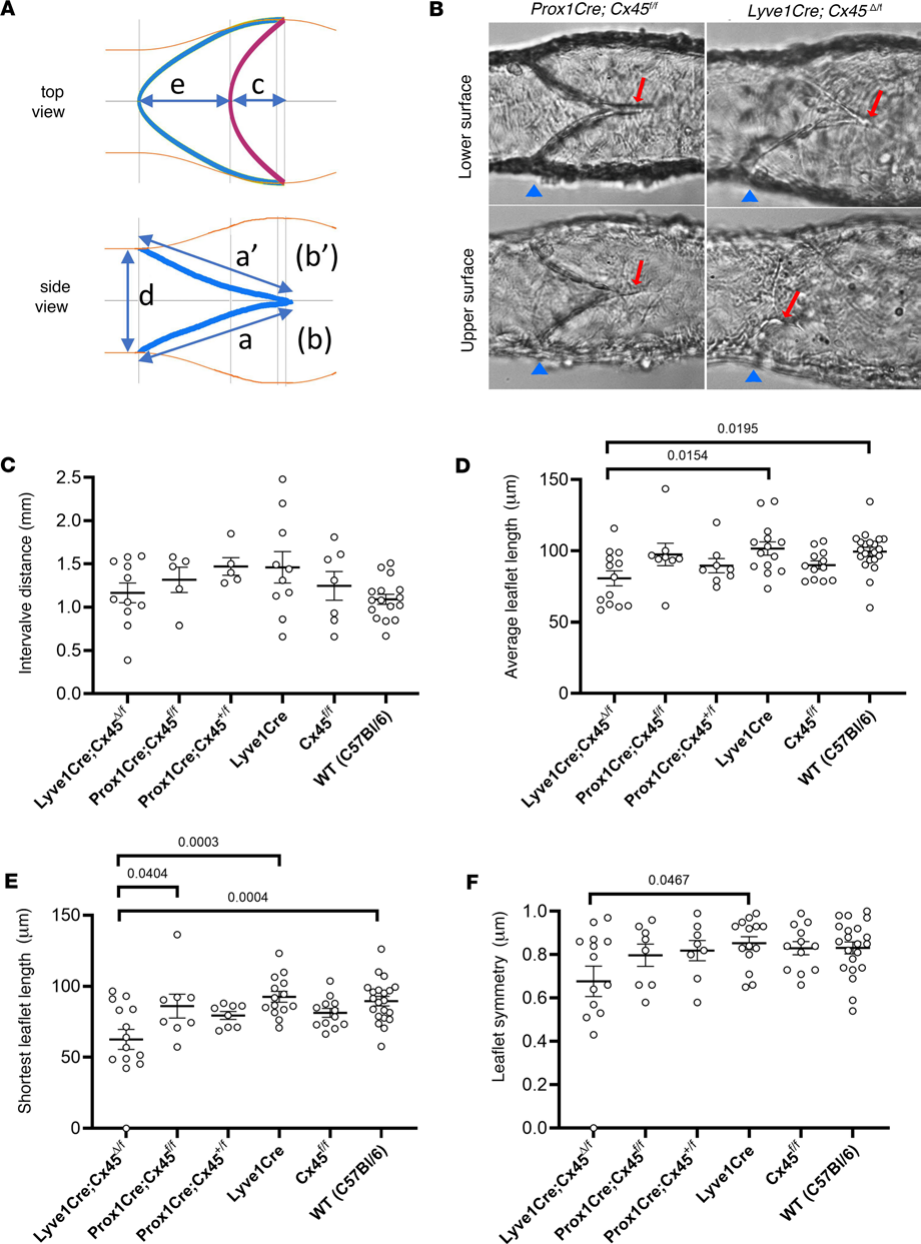
Valve dimension measurements made under bright-field microscopy during functional tests. (**A**) Schematic of a prototypical LV as viewed from the top or side; b and b’ are on the opposite surface of the valve sinus and are not shown. Neither are the commissures shown, but when present they extend downstream from the intersections of a and a’ and the intersections of b and b’. (**B**) Bright-field images of valves in *Prox1-CreER^T2^ Cx45^fl/fl^* and *Lyve1-Cre Cx45*^Δ/fl^ vessels, with arrows pointing to the downstream ends of the leaflets. (**C**) Intervalve distances in the 6 different genotypes of mice studied. (**D**–**F**) Measurements of average leaflet length (**D**), shortest leaflet length (**E**) and leaflet symmetry (**F**) in the 6 genotypes of mice. Significant differences were determined using 1-way ANOVAs with Dunn’s multiple-comparison post hoc tests to the respective “control” genotype. Nonsignificant comparisons are not marked. *WT* *N* = 16; *Cx45^fl/fl^ N* = 4; *Lyve1-Cre* *N* = 6; *Prox1-CreER^T2^ Cx45^fl/fl^ N* = 2; *Prox1-CreER^T2^ Cx45^+/fl^* *N* = 2; *Lyve1-Cre Cx45*^Δ/fl^ *N* = 8. The number of vessels for each group is reflected in the number of data points shown in the graphs.

Previously, leaflets in Rasa1-deficient lymphatic and venous valves were found to be significantly reduced in length (31), due to an inability of LV-forming LECs to export collagen IV, leading to the loss of leaflet LECs over time and eventual valve incompetency when a threshold number of leaflet LECs was exceeded (32). A similar analysis of Cx43-deficient valves also showed the same trend, with shorter leaflets in valves from *Lyve1-Cre Cx43*^Δ/fl^ mice compared with valves in *Cx43^fl/fl^* control mice (33). Here, we made similar measurements of leaflet dimensions under bright-field microscopy, as defined in Figure 6A, to test if they might reveal obvious structural differences that could explain back leak defects in *Lyve1-Cre Cx45*^Δ/fl^ valves. Ideally, the area of overlap between the 2 leaflet cusps in a closed valve would be the best predictor of valve competency, defined as the absence of back leak when closed. Although such measurements might be possible in confocal reconstructions, fixation potentially altered the valve properties, as demonstrated in Figure 1J. Under bright-field illumination, measurements of leaflet overlap were almost never possible in side views of the valves required for our functional tests. Likewise, measurement of the cusp length “e” or the actual length of the leaflet edge (the curved insertion paths of the leaflets from their common base to their tips) was impossible to make from side views. As an alternative, the best approximations of the leaflet lengths, a and a’ (as in ref. 31), were measured while focusing on the lower surface of the vessel, and then the corresponding approximations of leaflet lengths in the opposite wall (b and b’) were measured while focusing on the upper surface of the vessel. Images of an actual valve are shown in Figure 6B to illustrate the practical difficulties of such measurements.

Measurements of the number of valves per length of each cannulated vessel (before shortening to a single valve segment) did not reveal any significant differences in valve density among the 6 genotypes (Figure 6C). This result is consistent with the comparable mesenteric LV densities observed in *WT* and *Lyve1-Cre Cx45*^Δ/fl^ mice in Figure 3. However, there were significant differences in several of the leaflet dimensions between genotypes. Notably, significantly lower values of the leaflet length, the average of (a + a’)/2 and (b + b’)/2 (Figure 6, D and E), and a significantly lower degree of leaflet symmetry, the ratio of the 2 terms (Figure 6F), were evident in *Lyve1-Cre Cx45*^Δ/fl^ vessels, consistent with a possible contribution of these factors to back leak. Normalizing leaflet length to diameter (D) did not alter these conclusions. The difference between a and b for the *Lyve1-Cre Cx45*^Δ/fl^ valve in Figure 6B illustrates one example of the asymmetry observed in some *Cx45*-deficient valves. One other *Lyve1-Cre Cx45*^Δ/fl^ valve contained only a single leaflet, accounting for the lowest points in Figure 6, E and F. These results indicate that the *Lyve1-Cre Cx45*^Δ/fl^ mice had significantly shorter and asymmetric leaflets when compared with control littermates.

## Discussion

Our results demonstrate that Cx45, a previously unidentified LEC Cx, contributes to normal LV function. Cx45 expression is uniformly distributed in LECs of collecting lymphatic networks, in and across LVs. The distribution of Cx45 in lymphatic endothelium contrasts with that of Cx43, Cx37, and Cx47 (16) as Cx43 and Cx37 are differentially expressed on the upstream and downstream sides of the LV leaflets (26), and Cx47 is expressed only in a subset of valve cells (26). Searches of published single-cell RNA-Seq databases (34–36) independently validate Cx45 (*GJC1*) expression in LECs. The uniform axial distribution of Cx45 in lymphatic collectors suggests that its expression may be regulated in a different way than Cx37 and Cx43, perhaps independent of shear stress. Constitutive deletion of Cx45 from the lymphatic endothelium, using *Lyve1-Cre*, results in elevated back leak in 42% of popliteal LVs and an abnormally high adverse pressure gradient required for closure in 38% of popliteal LVs. Postnatal deletion of Cx45 after LVs have developed results in 21% of popliteal LVs with elevated back leak in *Prox1-CreER^T2^ Cx45^fl/fl^* valves. The absence of significant back leak or closure defects in *Prox1-CreER^T2^ Cx45^+/fl^* valves suggests that a single allele of Cx45 is sufficient to protect against valve defects. The leak of EBD from some *Lyve1-Cre Cx45*^Δ/fl^ vessels in vivo suggests that Cx45 may be required to maintain a normal LEC permeability barrier in collecting lymphatic vessels. It is interesting that dye leak only occurred in a subset of vessels, and usually over the entire visible length of the vessel, rather than being punctate as in some other genotypes with a disrupted permeability barrier (37, 38); this possibly may be related to higher intraluminal pressures in the leaky vessels. Future experiments with quantitative measurements of albumin or solute permeability under defined conditions (39, 40) would be valuable in confirming this finding and probing the underlying mechanism.

What is the relative importance of other Cx isoforms for valve function compared with Cx45? The only other functional data are from a previous study of *Lyve1-Cre Cx43^fl/fl^* popliteal LVs by Munger et al. (33), in which constitutive Cx43 deletion resulted in some degree of back leak in 67% (4 of 6) of LVs and complete incompetence in 17% (1 of 6) of LVs. In comparison, we find that 42% of LVs in *Lyve1-Cre Cx45*^Δ/fl^ popliteal vessels have some degree of elevated back leak and that 24% (4 of 17) are completely defective. These comparisons suggest somewhat comparable roles for Cx45 and Cx43 in the maintenance of mature, functioning LVs but with Cx43 deletion also affecting the total number of LVs that develop. The combination of back leak and closure defects in Cx45-deficient LVs implies that a fraction of those valves may never close under physiological conditions and that those which do close will permit varying degrees of backflow, resulting in inefficient lymph transport.

The mechanism by which Cx45 deficiency produces back leak remains unknown. Connexins enable the flux of ions and small molecules between cells and thus are more likely to play roles in LEC-LEC communication than in determining the structural integrity of cell-cell junctions. However, Cx45-mediated signaling might regulate the trafficking or localization of other junctional proteins such as occludins or cadherins that may be required at LEC-LEC junctions to maintain valve integrity. A model of LV leaflet behavior predicts that back leak is most likely to occur at the lateral margins where the leaflets insert into the wall (5). LEC-LEC junctions are particularly pronounced in these regions (Figure 1, B and E), and reinforcement may be needed to protect the valve against the unique mechanical forces associated with closure. Analyses of Claudin5 and VE-cadherin immunostaining patterns at low magnification in vivo did not reveal any obvious changes in the localization of these junctional proteins, but perhaps higher resolution confocal imaging would. We attempted to identify possible structural abnormalities in Cx45-deficient valves by measuring leaflet dimensions under bright-field microscopy during our functional tests, reasoning that such measurements could be used by others to guide identification of defective valves in the absence of functional tests. Confocal reconstructions of LEC fluorescence after subsequent fixation of the valves provided better resolution of the degree of leaflet overlap, but fixation itself altered the valve properties (Figure 1, I and J). Image analyses of unfixed valves indicated that constitutive Cx45 deficiency was associated with shorter leaflet lengths and increased leaflet asymmetry (Figure 6). Shorter valve leaflets have been previously shown in connection with Rasa1 deficiency (31) and Cx43 deficiency (33) and are predicted to decrease valve competency in one model of simulated valve function (41); however, valve asymmetry has not been proposed previously as a contributor to valve dysfunction, nor has this property been modeled. Shorter leaflet insertion paths on one side of the valve seemingly would decrease the amount of leaflet overlap on that side when the valve is closed, and thereby enhance the likelihood of back leak. The tendency for leaflets to be asymmetrical in *Lyve1-Cre Cx45*^Δ/fl^ vessels is suggestive of a disruption in the progression from stage 3 to stage 4 of valve development (42), in which the leaflet margins on one side have inserted into the wall and extended downstream, but the leaflet margins on the other side have not yet inserted into the opposite wall (43). Similarly, deletion of Cx45 from mature valves may induce reversion from stage 4 to stage 3. Whether it is possible to probe the sequence of events leading to leaflet asymmetry remains to be determined.

Although our valve tests were conducted ex vivo, the conditions used were physiologically relevant. Some studies have used backflow of injected dye in vivo as an indicator of defective lymphatic function (9, 26, 38), and our results from EBD injection protocols in Figure 3A indicate a quantitative and significant increase in the number of *Lyve1-Cre Cx45*^Δ/fl^ popliteal lymphatic afferents that exhibit backflow into side branches. However, these measurements depend on the volume of dye injection and the unknown pressure head generated during injection to ensure adequate lymphatic network perfusion. A brief period of backflow is known to occur during the lymphatic contraction cycle (44, 45), and backflow can occur during diastole in normal valves if a slight adverse pressure gradient (<0.3 cmH_2_O) is present and sufficient to drive retrograde flow but insufficient to produce closure (4). Thus, a valve might be characterized as abnormal in vivo, based on retrograde dye flux, even though it would fit our criteria for a normal valve (Figures 4 and 5) if studied ex vivo under defined pressures (i.e., with Pin and Pout known and spontaneous contractions blocked). Unlike EBD backflow protocols, the pressures used in ex vivo tests have been demonstrated to be physiologically relevant. Pressures in the mouse lymphatic system are not well documented, but recent measurements in mouse mesenteric lymphatic collectors indicate pressures of approximately 4 cmH_2_O in control mice and approximately 15 cmH_2_O in mice with partial lymphatic obstruction (46). As the distance from the heart to the lower leg is about 5 cm in an adult mouse (23, 47), lymph would need to be transported against a standing column of fluid up to ~5 cmH_2_O if a mouse with incompetent LVs were oriented in the vertical position (i.e., with LVs unable to disrupt the column of lymph). Our typical Pout ramp protocol to 10 cmH_2_O is required to detect low levels of back leak for subtle defects characteristic of *Foxc2^+/fl^* and *Prox1-Cre Foxo1^fl/fl^* mice (11, 12), but in Cx45-deficient vessels we detected significant back leak at Pout levels well below 10 cmH_2_O (Figure 4L). Closure tests were conducted at multiple pressures/diameters below and including 10 cmH_2_O, and the ΔP for closure at Pin = 0.5 cmH_2_O was chosen as a representative pressure for statistical tests between genotypes (Figure 5O). Importantly, valves with a ΔP for closure greater than 30 cmH_2_O, such as those shown in Figure 5N, would be predicted to never close, even in contracting vessels.

Finally, although there are no published reports of human lymphedema associated with mutations in Cx45 (*GJC1*), this Cx is critically important for a number of other physiological systems, most notably for the conduction of electrical signals in the developing heart (48, 49). Loss-of-function mutations in Cx45 cause familial atrial fibrillation and conduction disease in humans (50, 51), and global deletion of *Cx45* is lethal in mice (52, 53). Because of the importance of Cx45 in the developing cardiac conduction system, it is likely that most human loss-of-function mutations in *Cx45* are lethal so that defects in LVs that could potentially produce lymphedema would not be detected.

In conclusion, we have shown that Cx45, in addition to being expressed in LMCs, is functionally expressed in LECs throughout collecting lymphatic networks in mice and is required, along with other LEC Cx isoforms, for the development and maintenance of competent LVs.

## Methods

### Sex as a biological variable.

Our study examined male and female animals, and similar findings are reported for both sexes.

### Mice.

*Cx45^fl/fl^* mice (25) were a gift from Klaus Willecke, University of Bonn, Bonn, Germany. *Prox1-CreER^T2^* mice (2) were a gift from Taija Mäkinen, Uppsala University, Uppsala, Sweden. *Prox1-GFP* mice (54) were a gift from Young-Kwon Hong, University of Southern California, Los Angeles, California, USA. *Cx45^fl/fl^*, *Lyve1-GFP-Cre*, *Prox1-GFP*, and *Prox1-CreER^T2^* mice were backcrossed to *WT* mice (C57BL/6J, strain 000664, Jackson Laboratory) for at least 6 generations. *Lyve1-GFP-Cre* mice express EGFP under the *Lyve1* promoter, but EGFP expression is not affected by recombination; therefore, these mice are referred to as *Lyve1-Cre* mice. For genotyping, genomic DNA was extracted from tail clips using the HotSHOT method. Genotypes were determined by PCR with 2xM-PCR OPTI Mix (catalog B45012, Bimake) according to the provider’s instructions. Mice of both sexes were studied.

To delete Cx45 embryonically, male *Lyve1-Cre^+/fl^* mice were bred with female *Cx45^+/fl^* mice, and progeny that carried a germ line deletion of 1 Cx45 allele are designated as *Lyve1-Cre Cx45*^Δ/fl^ mice. Alternative matings (female *Lyve1-Cre^+/fl^* mice with male *Cx45^fl/fl^* mice) produced some *Lyve1-Cre Cx45^fl/fl^* mice of both sexes. To delete Cx45 postnatally, we bred *Prox1-CreER^T2^ Cx45^fl/fl^* mice and induced them with 5 consecutive daily i.p. injections of 100 mg tamoxifen (10 mg/kg; in safflower oil) beginning at P30. The deletion of Cx45 accomplished by Cre-recombinase activity led concomitantly to the expression of the reporter EGFP (25). Mice were studied 2–3 weeks later. Mice were studied at E18.5 and P10 for mesenteric valve counting and at P42–P126 for valve function tests.

### Vessel isolation and cannulation.

Mice were anesthetized with ketamine/xylazine (100/10 mg/kg, i.p.) and placed in the prone position. The dissection and cannulation of popliteal and mesenteric lymphatic vessels was performed as described previously (37, 55). After cannulation, the stage containing the vessel, chamber, pipette holders, and micromanipulators was moved to an inverted microscope (modified Zeiss ACM). The backs of the micropipettes were connected to low-pressure transducers and a computerized pressure controller (56), allowing independent control of inflow (Pin) and outflow (Pout) pressures. The vessel axial length was adjusted with pressures briefly set to 10 cmH_2_O. Custom LabVIEW programs (National Instruments) acquired analog data from the pressure transducers simultaneously with vessel inner diameter, as detected from video images acquired using a Basler firewire camera (56, 57).

### Valve function tests.

Luminal pressure on the inflow side of the valve (Psn) was measured with a servo-null micropipette inserted through the wall. An initial hole was made with a pilot micropipette, which was then removed and replaced with a servo-null micropipette (tip diameter = 3–5 μm). After insertion, the servo-null micropipette was advanced to seal the hole. The calibration of the servo-null pipette was checked, and adjusted as needed, after raising Pin and Pout simultaneously between 0.5 and 10 cmH_2_O. Back leak tests and valve closure tests were performed as described previously (37). To ensure accurate and consistent measurements of valve back leak, 1) all 3 transducers (Pin, Psn, Pout) were calibrated before each experiment; 2) the same pair of cannulation pipettes was used for all experiments to maintain consistent pipette resistances; 3) the pipettes were cleaned after each experiment with boiling water and acetone and checked before each valve test to ensure that the tips were free of debris (if needed, debris was cleared by sliding 14 μm suture several times through the tip); 4) the lines were free of bubbles; and 5) the Psn pipette calibration was rechecked at the end of the valve test.

After function tests were completed, some vessels were fixed in 1% PFA for 5–10 minutes at room temperature while pressurized, then fixed overnight in 1% PFA at 4°C, followed by repeated rinsing with and storage in PBS containing 0.1% sodium azide, for subsequent immunostaining.

### Solutions and chemicals.

Krebs buffer contained 146.9 mM NaCl, 4.7 mM KCl, 2 mM CaCl_2_·2H_2_O, 1.2 mM MgSO_4_, 1.2 mM NaH_2_PO_4_·H_2_O, 3 mM NaHCO_3_, 1.5 mM Na-HEPES, and 5 mM d-glucose (pH = 7.4). A buffer of the same composition (“Krebs-BSA”) also contained 0.5% bovine serum albumin. Krebs-BSA buffer was present both luminally and abluminally during cannulation, with the abluminal solution constantly exchanged with Krebs during the experimental protocol. For Ca^2+^-free Krebs, 3 mM EGTA replaced CaCl_2_·2H_2_O. Purified BSA was obtained from US Biochemicals, MgSO_4_ and Na-HEPES were obtained from Thermo Fisher Scientific, and all other chemicals were obtained from MilliporeSigma except as otherwise specified.

### Confocal imaging.

Fixed lymphatic vessels were washed in PBS 3 times, permeabilized with a 0.1% Triton X-100 solution in PBS for 1 hour, blocked for nonspecific binding using 5% donkey serum (MilliporeSigma, catalog D9663) in PBS for 2 hours at 4°C, and then incubated at 4°C overnight with primary antibodies. The antibodies were anti-GFP (1:300) (Invitrogen A-11122) and anti-Cd144 (VE-cadherin) (1:500) (BD Pharmingen 550548) in a PBS solution containing 5% donkey serum. Vessels were washed for 2–4 hours in PBS at 4°C, exchanging the PBS 3–4 times over the washing period. Vessels were then incubated overnight with secondary antibodies (donkey anti-rabbit and donkey anti-rat at 1:500 dilution, BD Pharmingen A21206 and A48272, respectively) at 4°C. The vessels were washed with PBS for 2 hours on a rocker platform, with the PBS solution replaced every ~30 minutes, then incubated in a DAPI solution (Thermo Fisher Scientific catalog EN62248 1 mg/mL stock and used at 1:1,000 dilution) for 15 minutes at room temperature. Finally, the vessels were transferred to a myography chamber, where they were recannulated and pressurized. The chamber was custom made. Fluorescence image stacks were acquired on a Leica DMI8 inverted fluorescence microscope using an Andor Dragonfly 202 high-speed confocal imaging platform equipped with Borealis enhanced illumination (40 μm pinhole disk). Images were acquired with a Zyla PLUS 4.2 Megapixel sCMOS camera and an HCX PL APO 40×/1.10W CORR objective using excitation from 405, 488, and 647 nm solid-state diode laser lines. *Z* axis image stacks were acquired in 0.24 μm intervals, then processed and rendered using Imaris x64 9.7.2 as 3-dimensional orthographic isometric projections.

### Immunohistochemistry.

Immunohistochemistry (IHC) was performed according to detailed protocols published recently (29). A modified iDISCO protocol (58) was used for whole-mount sections of the dorsal skin and mesentery. For staining with VE-cadherin antibody, a previously described whole-mount IHC protocol was used (29). Primary antibodies for IHC included rabbit anti-PROX1 (catalog 11-002, Angiobio), rat anti–mouse CD31 (catalog 553370, BD Pharmingen), rat anti–mouse VE-cadherin (catalog 550548, BD Pharmingen), chicken anti-GFP (catalog ab13970, Abcam), and rabbit anti–mouse CLDN5 (catalog 34-1600, Thermo Fisher Scientific). Goat anti–human PROX1 (catalog AF2727), goat anti–mouse VEGFR3 (catalog AF743), and goat anti–mouse ITGA9 (catalog AF3827) were from R&D Systems, Bio-Techne. Secondary antibodies for IHC included Cy3-conjugated donkey anti-rabbit, Cy3-conjugated donkey anti-goat, Cy5-conjugated donkey anti-rat, FITC-conjugated donkey anti-chicken, Alexa Fluor 488–conjugated donkey anti-goat, and Alexa Fluor 488–conjugated donkey anti-rabbit antibodies, purchased from Jackson ImmunoResearch Laboratories (catalog 711-165-152, 705-165-147, 712-175-150, 703-095-155, 705-545-147, and 711-547-003, respectively). Alexa Fluor 488–conjugated donkey anti-rat antibody was purchased from Invitrogen (catalog A-21208).

### Quantification of mesenteric LVs.

Mesenteric LVs were identified by high PROX1 staining of collectors from the mesenteric lymph nodes to the point where the major collecting vessels branched into the gut wall. Staining was quantified using a Nikon Eclipse 80i microscope equipped with an Andor camera and Nikon NIS Elements Software. For E18.5 embryonic mesentery, the total number of valves were counted on 2 vessels per mesentery, and the average number was presented for each animal. Four embryos were analyzed for each genotype. For P10 mesentery, the total numbers of valves were normalized to the vessel length. Four vessels from each mesentery were quantified, and 4 animals were analyzed for each genotype. To determine the vessel diameter, 5 locations were measured along a single mesenteric vessel, and the average of the 5 measurements was presented for each animal. For E18.5 embryonic mesentery, 2 vessels were measured for each animal, and 4 animals were analyzed for each genotype. For P10 pups, 4 vessels were measured for each animal, and 4 animals were analyzed for each genotype. Measurements were made using NIS Elements Software.

### FACS analysis.

Inguinal-axillary lymphatic vessels from *Prox1-GFP* mice or tamoxifen-treated *Prox1-CreER^T2^ Cx45^fl/fl^* mice were dissected and cleaned of fat and connective tissue. Cleaned vessel segments were digested into single cells as described previously (59). LECs expressing EGFP were sorted by FACS using a Beckman-Coulter MoFlo XDP instrument with excitation laser (488 nm) and emission filter (530 ± 40 nm), a 70 μm nozzle, a sheath pressure of 45 psi, and a sort rate of 100 events per second. Sorting was performed at the Cell and Immunobiology Core facility at the University of Missouri.

### RNA isolation, reverse transcriptase PCR, endpoint PCR, and qPCR.

Total RNA was extracted from FACS-sorted cells and endpoint PCR performed as described previously (59). All primers (listed in Table 1) were designed to amplify intron-spanning DNA regions. qPCR was performed on cDNA prepared from each sample using 2× PrimeTime Gene Expression Master Mix (Integrated DNA Technologies) with predesigned TaqMan probes (Integrated DNA Technologies), as listed in Table 2. qPCR protocols were as follows: preheating to 95°C for 3 minutes, 45 cycles of 2-step cycling of denaturation at 95°C for 15 seconds, and annealing/extension steps of 30 seconds at 60°C. Data collection was carried out using a Bio-Rad CFX 96 Real-Time Detection System (software version Bio-Rad CFX Manager 3.1; Bio-Rad). For analyses, the results were expressed as a ratio of target gene/reference gene (β-actin).

### In vivo tests of backflow.

An anesthetized mouse was placed face down on a heating pad, and each hind limb was extended and held in place with a piece of tape. A small amount (~5–10 μL) of EBD (1% in sterile saline) was injected into the dermis of the foot pad using a 30-gauge needle. Typically, the filling of both popliteal afferents in each leg required that half the dye volume be injected into the top of the foot and the other half into the side of the foot. The skin over the 2 primary popliteal afferents in the thigh region was then opened, retracted, and covered with Krebs-BSA. Any loose connective tissue or fat was removed to ensure maximum visibility of the popliteal afferents. Images of the popliteal network were collected using a Leica S9i dissecting microscope (original magnification, 10×–40×) with built-in camera and stored on an SDHC card. Successful EBD injection was confirmed by filling of both afferent vessels and the popliteal nodes. If needed, gentle pressure was applied to the injection site with a cotton swab to facilitate filling. Dye backflow into side branches was monitored in real time and documented with photos. In each case of backflow we confirmed that EBD was flowing from one of the main vessels backward into a side branch, rather than in the other direction. The procedure was then repeated for the other leg. Popliteal afferents that did not completely fill with EBD were not counted in the analysis.

### Statistics.

Microsoft Excel was used to compile the initial data and to calculate back leak pressures (Pout–Psn) and values of ΔP for closure (Pout–Pin). Igor (Wavemetrics) was used for the display of representative traces. LabVIEW was used to read raw data from text files and bin that data into discrete Pout intervals for subsequent statistical analysis. Prism v9 (GraphPad) was used for summary plots and statistics. Specific statistical tests for the various data sets are described in the respective figure legends. The normality of each data set was tested prior to using ANOVA and, if normality was not satisfied in a majority of the standard Prism normality tests, then nonparametric tests (e.g., Kruskal-Wallis or Mann-Whitney *U* tests) were used instead of ANOVAs or *t* tests. When not explicitly stated, *P* values less than 0.05 are considered statistically significant.

### Study approval.

All procedures were approved by the institutional review boards at the University of Missouri, Oklahoma Medical Research Foundation, and Tulane School of Medicine and complied with the standards stated in the *Guide for the Care and Use of Laboratory Animals* (NIH, National Academies Press, revised 2011).

### Data availability.

The data sets shown in all the figures are listed in an associated spreadsheet of Supporting Data Values. LabVIEW code for data collection and analyses is available at https://doi.org/10.5281/zenodo.8286107 and https://doi.org/10.5281/zenodo.8286119

## Author contributions

MJD and RSS designed the experiments. MJD, XG, JACG, SDZ, and ML performed the experiments and analyzed the results. MJD, AMS, and RSS drafted the manuscript. All authors edited the manuscript and approved the final version.

## Supplementary Material

Supplemental data

Unedited blot and gel images

Supplemental video 1

Supplemental video 2

Supporting data values

## Figures and Tables

**Table 1:**
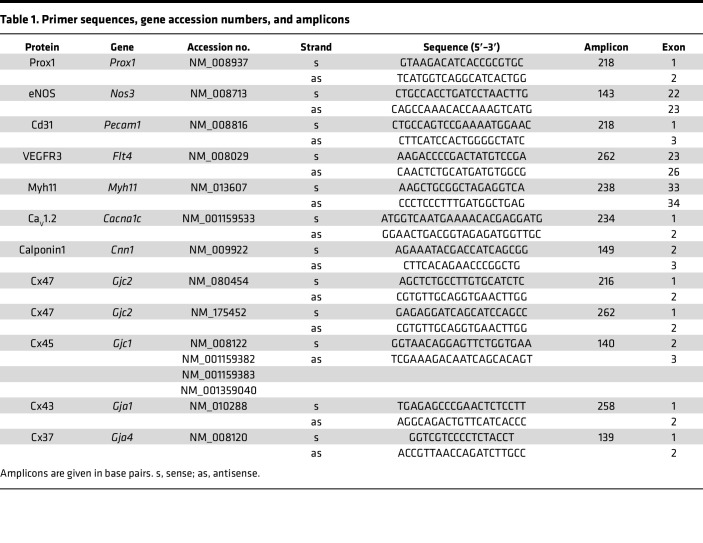
Primer sequences, gene accession numbers, and amplicons

**Table 2:**
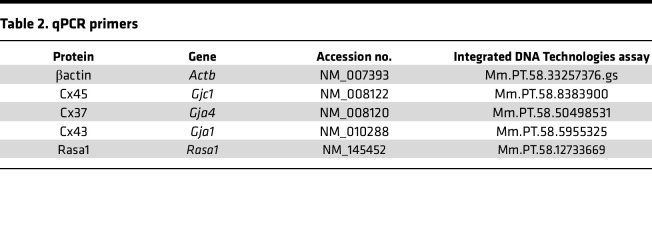
qPCR primers

## Version 1 07/29/2024

In-Press Preview

## Version 2 08/22/2024

Electronic publication
