# Supplementary material for: Connexin-45 is expressed in mouse lymphatic endothelium and required for lymphatic valve function
Source: JCI Insight. 2024 Jul 18;9(16):e169931. doi: 10.1172/jci.insight.169931 (PMC11343601; doi:10.1172/jci.insight.169931)
Supplement: Supplemental data [file jciinsight-9-169931-s070.pdf]

Suppl. Fig 1.

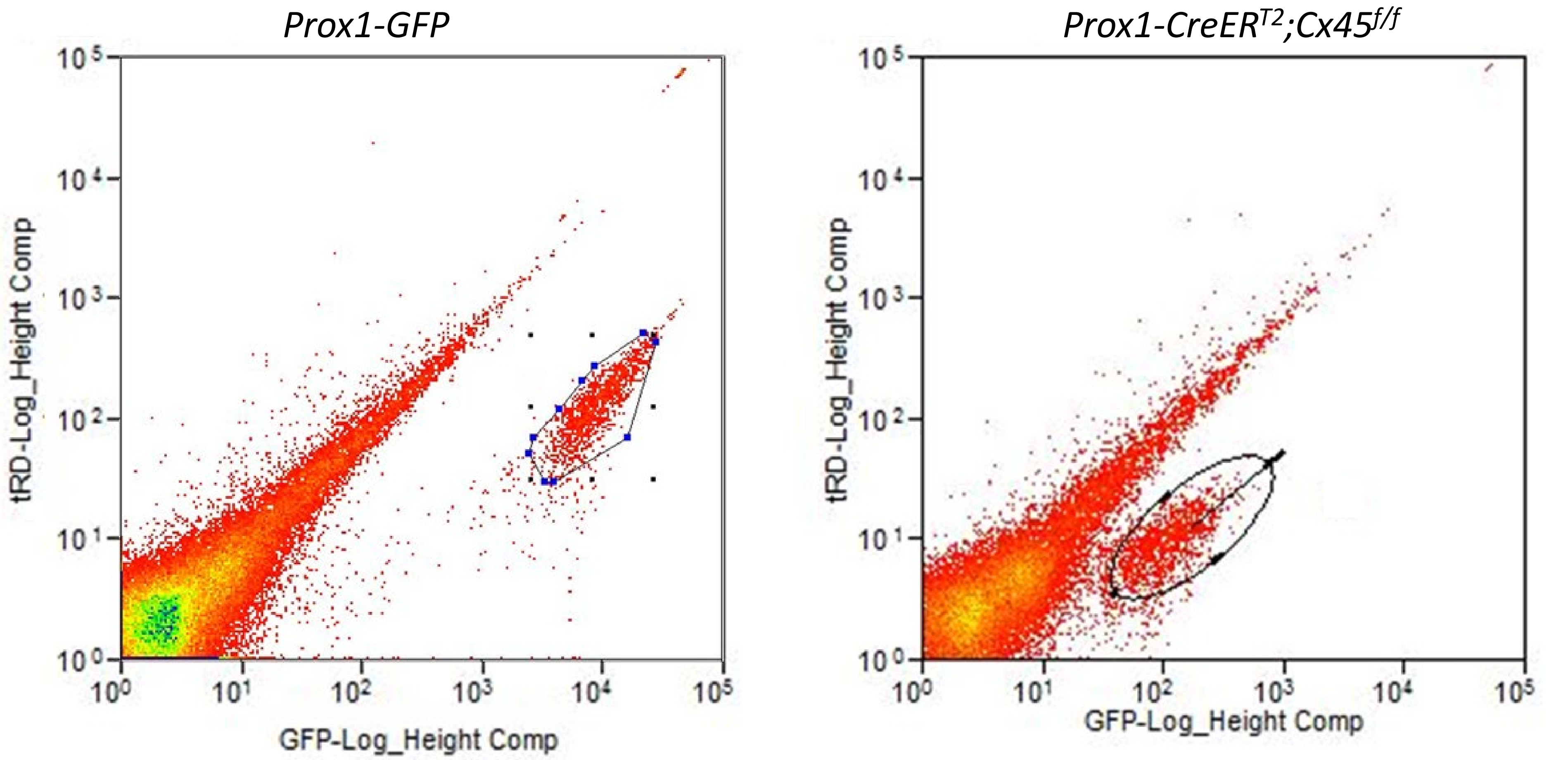

Suppl. Fig. 1 legend. FACS analysis. FACS analysis of GFP+ cells sorted from *Prox1-GFP* and *Prox1-CreER*^{T2}*;Cx45*$^{f/f}$ vessels, with sorting windows circled.

Suppl. Fig 2.

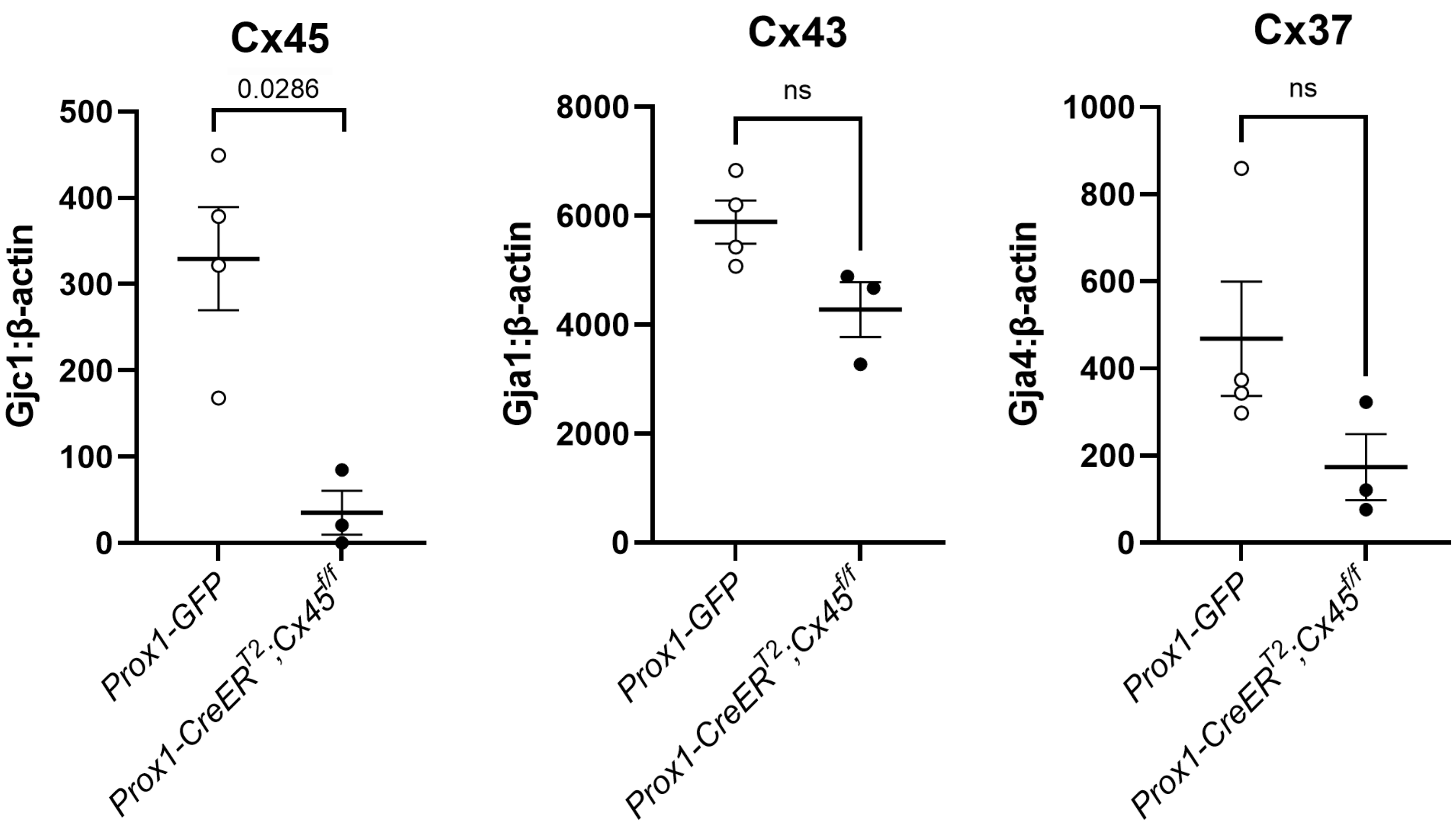

Suppl. Fig. 2 legend. qPCR analysis reveals no significant changes in *Cx43* or *Cx37* mRNA levels after *Cx45* deletion. qPCR analysis of Cx isoform mRNA levels in GFP+ cells sorted from *Prox1-GFP* and *Prox1-CreER^{T2};Cx45$^{f/f}$* vessels. Comparisons between *Prox1-GFP* and *Prox1-CreER^{T2};Cx45$^{f/f}$* groups were made using Mann-Whitney U tests; * = $p<0.05$.

Suppl. Fig 3.

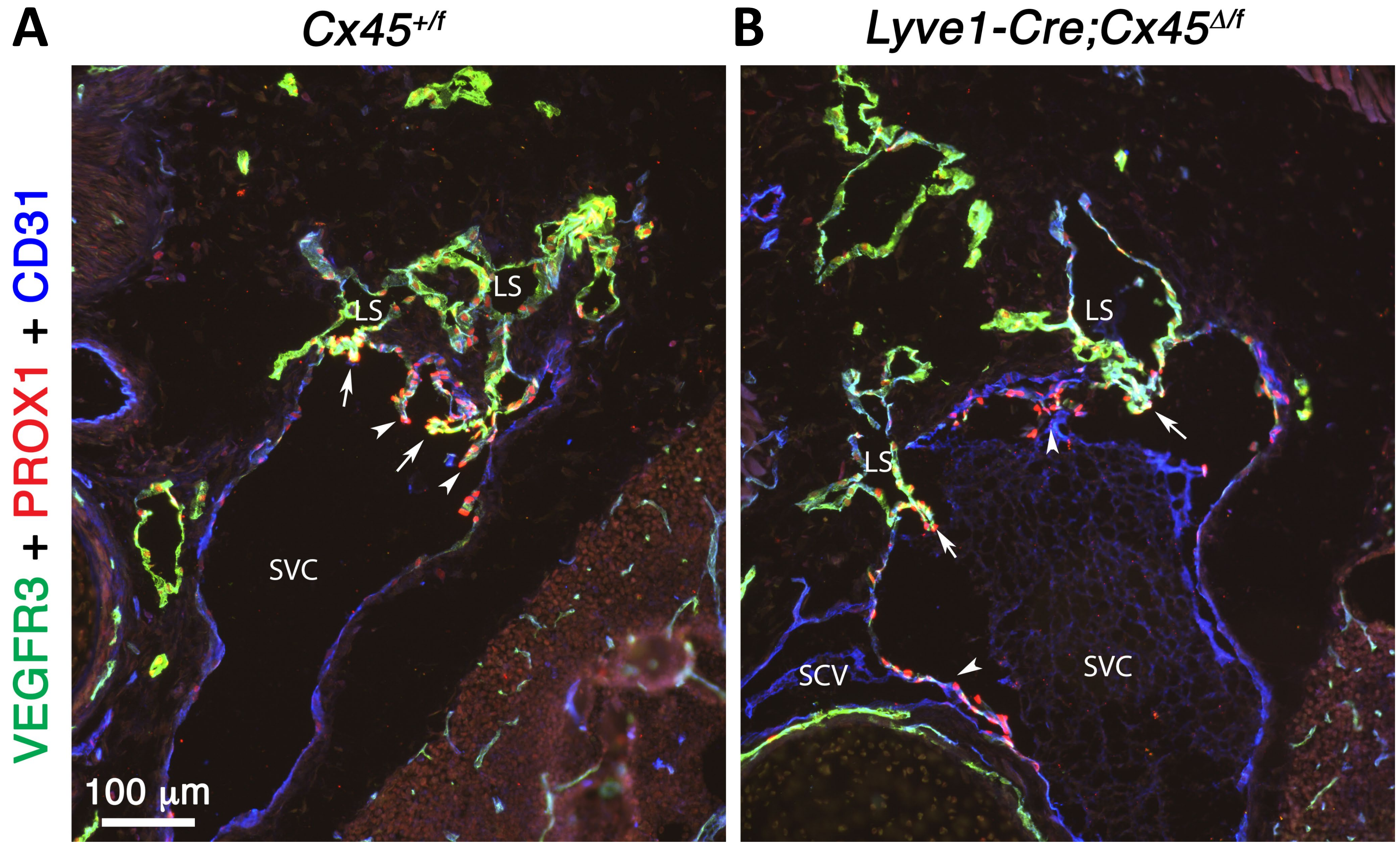

Suppl. Fig. 3 legend. Deletion of Cx45 does not interfere with the development of lymphovenous valves (LVVs). Venous and lymphovenous valves were unaffected by the deletion of *Cx45*. **A**) E18.5 *Cx45*$^{+/f}$ and **B**) *Lyve1-Cre;Cx45*$^{\Delta/f}$ embryos were frontally sectioned and the junctions of the jugular (JS) and subclavian veins (SCV) were analyzed using the indicated antibodies. SVC= superior vena cava. No obvious defects were observed in the lymphovenous valves (arrows) or the venous valves (arrowheads) of *Lyve1-Cre;Cx45*$^{\Delta/f}$ mice. Images in each panel are representative of 4 experiments.
