# Supplementary figures and images for: Connexin-45 is expressed in mouse lymphatic endothelium and required for lymphatic valve function
Source: JCI Insight. 2024 Jul 18;9(16):e169931. doi: 10.1172/jci.insight.169931 (PMC11343601; doi:10.1172/jci.insight.169931)

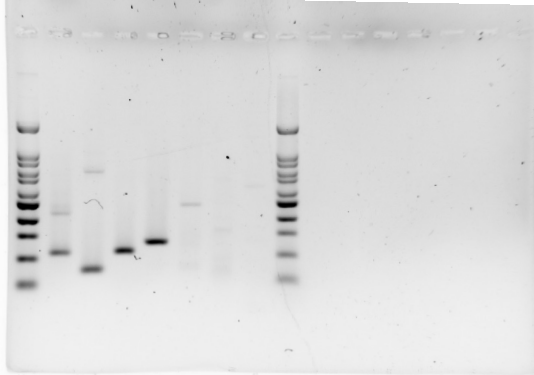

Fig 2A

Fig 2B

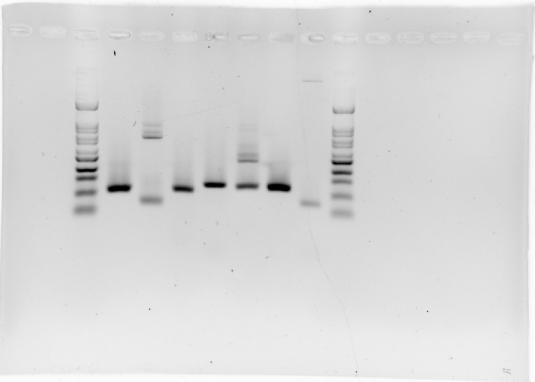

Fig 2C

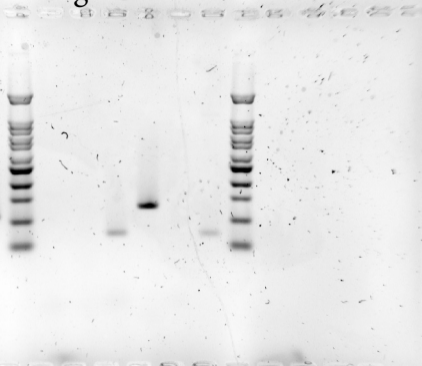

Fig 2D

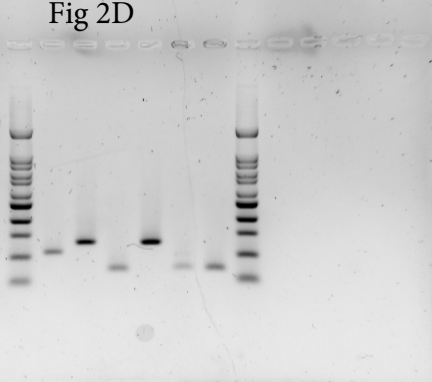

Fig 2E-F

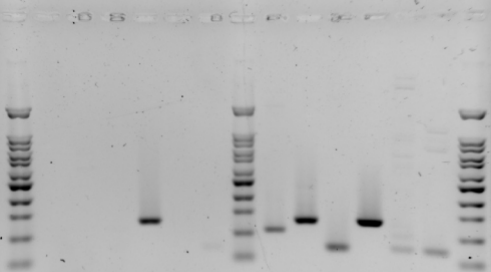

Supplement: Unedited blot and gel images [file jciinsight-9-169931-s071.pdf]
